# Outcomes of people with TB reported to the WHO Global Clinical Platform of COVID-19

**DOI:** 10.5588/ijtldopen.24.0210

**Published:** 2024-08-01

**Authors:** M. Bastard, D. Falzon, S. Bertagnolio, R. Silva, S.S. Thwin, C. Siquiera Boccolini, J. Rylance, J. Diaz, M. Zignol

**Affiliations:** ^1^ Global Tuberculosis Programme,; ^2^ Department of Antimicrobial Resistance,; ^3^ Department of Sexual and Reproductive Health and Research, and; ^4^ Health Emergencies Programme, Department of Country Readiness Strengthening, World Health Organization, Geneva, Switzerland

**Keywords:** tuberculosis, COVID-19, SARS-CoV-2, mortality, disease severity, hospitalisation, clinical database

## Abstract

**BACKGROUND:**

TB is a leading infectious cause of death worldwide. The COVID-19 pandemic raised concerns that the burden of TB disease and death would increase due to the synergy between the two conditions.

**METHODS:**

We used individual-level data submitted to the WHO Global Clinical Platform for COVID-19 on hospitalised patients to explore associations of TB with mortality using multivariable logistic regression.

**RESULTS:**

Data were available from 453,233 persons with COVID-19 and known TB status and mortality outcomes from 62 countries (96% SARS-CoV-2 test-positive). Of these, 48% were male, and the median age was 53 years (IQR 38–67). There were 8,214 cases with current TB reported by 46 countries, mainly from Africa. Of people with current TB, 31.4% were admitted with severe illness, and 24.5% died. Current TB was independently associated with higher mortality when adjusted for age, sex, HIV status, illness severity at hospital admission, and underlying conditions (adjusted RR 1.47, 95% CI 1.35–1.61).

**CONCLUSION:**

Current or past TB were independent risk factors for in-hospital mortality regardless of illness severity at admission. Caveats for interpretation include changes during the data collection period (viral variation, vaccination coverage) and opportunistic sampling. However, the platform exemplifies how timely, coordinated global reporting can inform our understanding of health emergencies and the vulnerable populations affected.

## RÉSUMÉ

**CONTEXTE:**

La TB est l'une des principales causes infectieuses de décès dans le monde. La pandémie de COVID-19 a fait craindre que le fardeau de la TB et des décès n'augmente en raison de la synergie entre les deux maladies.

**MÉTHODES:**

Nous avons utilisé les données individuelles soumises à la Plateforme clinique mondiale de l'OMS pour la COVID-19 sur les patients hospitalisés pour explorer les associations entre la TB et la mortalité à l'aide d'une régression logistique multivariée.

**RÉSULTATS:**

Des données étaient disponibles sur 453 233 personnes atteintes de COVID-19 et connues pour le statut de TB et les résultats de mortalité dans 62 pays (96% de tests positifs au SRAS-CoV-2). Parmi eux, 48% étaient des hommes et l'âge médian était de 53 ans (IQR 38–67). Un total de 8 214 cas de TB ont été signalés par 46 pays, principalement en Afrique. Parmi les personnes atteintes de TB actuelle, 31,4% ont été admises avec une maladie grave et 24,5% sont décédées. La TB actuelle était indépendamment associée à une mortalité plus élevée lorsqu'elle était ajustée en fonction de l'âge, du sexe, du statut VIH, de la gravité de la maladie à l'admission à l'hôpital et des affections sous-jacentes (RR ajusté 1,47 ; IC à 95% 1,35–1,61).

**CONCLUSION:**

La TB actuelle ou passée était un facteur de risque indépendant de mortalité à l'hôpital, quelle que soit la gravité de la maladie à l'admission. Les mises en garde concernant l'interprétation comprennent les changements au cours de la période de collecte des données (variation virale, couverture vaccinale) et l'échantillonnage opportuniste. Cependant, la plateforme illustre comment des rapports mondiaux opportuns et coordonnés peuvent éclairer notre compréhension des urgences sanitaires et des populations vulnérables touchées.

By the end of May 2024, over 775.4 million COVID-19 cases and 7 million COVID-19 deaths had been reported globally.^1^ Low-income and middle-income countries (LMICs), notably Brazil, India, and South Africa, have reported high numbers of COVID-19 cases. In 2020, COVID-19 replaced TB as the leading single infectious cause of death worldwide: it is estimated that in 2022, about 10.6 million new TB cases emerged, and 1.3 million TB deaths occurred globally.^2^ Over 60% of the global burden of TB is concentrated in populous Asian countries and over 20% in African countries. Global TB incidence and TB mortality increased during the first years of the pandemic, probably as a result of social and health system disruptions caused by COVID-19.

Given that in most TB patients, the primary localisation of the disease is in the lungs, the advent of COVID-19 led to concerns that the burden of TB disease and death would increase due to the synergy between the two conditions, particularly in areas with limited resources and healthcare services. The TB epidemic is strongly influenced by broader social and economic development as well as risk factors more especially linked to health, such as alcohol use disorders, diabetes, HIV infection, smoking, and undernourishment. Early analysis of evidence suggested that both current and past TB increased the risk of dying in COVID-19 patients and that older age and diabetes were also associated with poorer outcomes in COVID-19 as in TB.^3^ In many high TB burden settings, access to the COVID-19 vaccine was minimal, and vaccine prioritisation for vulnerable subpopulations was therefore critical.

Disease modelling described that a putative 25% reduction in TB detection for 6 months due to COVID-19-related disruptions would lead to a 26% increase in TB deaths, negating all progress made since 2012.^4^ This scenario was consistent with WHO data, which showed substantial reductions in TB notification sustained over successive months in 2020 in many high TB-burden countries as COVID-19 measures were introduced. In 2020 and 2021, disruptions associated with the COVID-19 pandemic did, in fact, have a substantial impact on TB case notifications.^5–7^ Globally, new TB notifications reported to the WHO fell by 18% from 7.1 million in 2019 to 5.8 million in 2020. This increased to 7.5 million new TB notifications in 2022, showing that there has been a good post-COVID recovery in access to and provision of health services in many (but not all) countries. This may reflect, at least in part, a backlog of delayed TB diagnoses related to pandemic-associated disruption.

Identifying risk factors for severe or fatal COVID-19 can help public health authorities prioritise populations and preventive interventions. WHO created the Global Clinical Platform on COVID-19 shortly after the pandemic was declared in April 2020.^8^ This platform consolidates anonymised individual-level clinical data of hospitalised patients with suspected or confirmed COVID-19 from health facilities worldwide. We used the data from this platform to explore the association between TB disease—past and current—and severe or fatal outcomes in hospitalised COVID-19 patients.

## METHODS

### Data sources

Data were contributed by Ministries of Health, research networks, and health facilities using a standardised Case Report Form (CRF) and data dictionary.^8^ Additional data were solicited from research networks, health facilities, and authors of published articles identified by continued scanning of literature and imported from their existing datasets. The platform allowed retrospective or prospective data collection.

The standardised set of variables described patient data at hospital admission, daily review, and hospital discharge. Each country dataset included demographics, pregnancy status, vital signs, anthropometrics, past medical and medication history, clinical features, laboratory test results, therapeutics, use of oxygen, use of mechanical ventilation, complications arising due to COVID-19, and clinical outcomes (discharge, death, transfer to another facility, intensive care admission, and ongoing hospitalisation).

### Study design and population

All patients admitted to a healthcare facility with laboratory-confirmed or clinically suspected COVID-19 from 01 January 2020 to 31 May 2023 were eligible for inclusion. Cases were defined as mild/moderate or severe/critical according to a modified definition from the WHO Clinical Management Guidelines of COVID-19.

### Statistical analysis

Patient characteristics were described by current or past TB disease status. Missing data were presented as counts and excluded from the calculation of proportions. Length of stay data were presented with medians and interquartile ranges (IQRs) and compared with the Wilcoxon rank-sum test. In-hospital deaths were defined as deaths occurring during hospitalisation. Case-fatality ratio (CFR) was calculated from the number of in-hospital deaths as a proportion of people dying in the hospital plus those discharged alive. Patients with other outcomes (palliative discharge, still hospitalised and transfer)—which represent 2.5% of admissions—were excluded from the CFR calculation. Multivariable log-binomial regression was used to evaluate the independent association of current TB with in-hospital mortality, using adjusted risk ratios (aRRs) and 95% confidence intervals (95% CIs). A *P*-value under 0.05 was considered statistically significant. The estimation of variance in the model was adjusted for potential clustering at the country level. The following factors were a priori included in the model: age, sex, severity of COVID-19 disease, HIV status, and other comorbidities (chronic cardiac disease, diabetes, hypertension, chronic pulmonary disease, and asthma). Sensitivity analysis was done by including the history of TB disease in the final model, with stratification by HIV status to explore the effect modification of mortality risk. Analyses were conducted in R v4.3.1 (R Computing, Vienna, Austria).^9^

### Ethics clearance

The analysis described in this paper conforms to the Platform's analysis plan. This plan was exempted from ethics review clearance by the WHO Research Ethics Review Committee (ERC; Geneva, Switzerland) because we used anonymised clinical surveillance data. Ethics clearance was obtained, where necessary, by institutional or national bodies contributing data.

## RESULTS

### Patient characteristics

**Figure 1.**
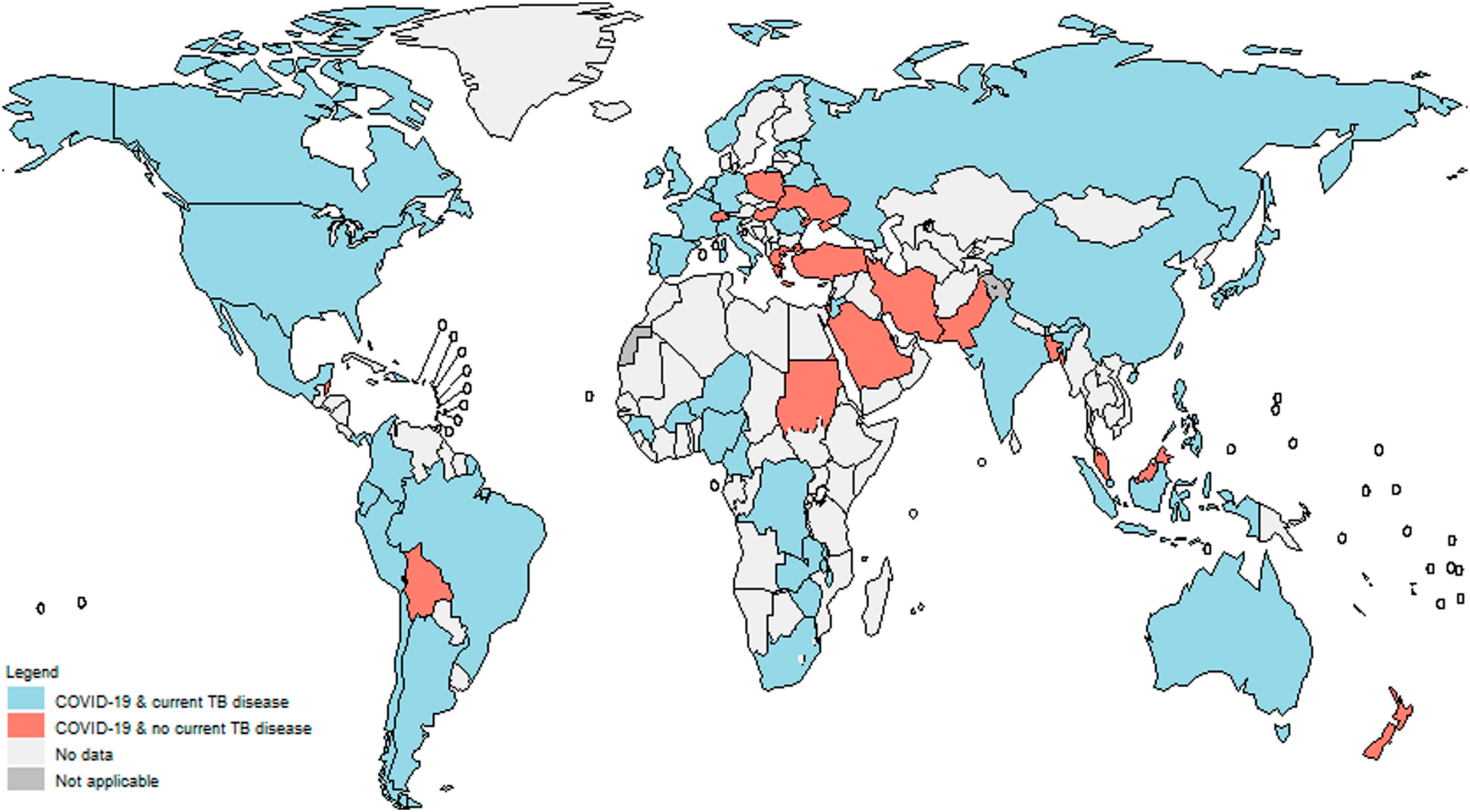
Countries contributing clinical data on TB among hospitalised COVID-19 cases to the WHO Global Clinical Platform for COVID-19. * 62 countries contributing clinical data on people with current TB hospitalised with COVID-19: Argentina, Australia, Bangladesh, Belarus, Belgium, Belize, Bolivia (Plurinational State of), Brazil, Burkina Faso, Cameroon, Canada, Chile, China, Colombia, Democratic Republic of Congo, Dominican Republic, Ecuador, Estonia, France, Gambia, Germany, Greece, Guinea, Hungary, India, Indonesia, Iran (Islamic Republic of), Ireland, Israel, Italy, Japan, Jordan, Kuwait, Malawi, Malaysia, Mexico, Netherlands, New Zealand, Niger, Nigeria, Norway, Pakistan, Panama, Peru, Philippines, Poland, Portugal, Republic of Korea, Romania, Russian Federation, Saudi Arabia, Singapore, South Africa, Spain, Sudan, Switzerland, Türkiye, Ukraine, United Kingdom, United States of America, Zambia, Zimbabwe. The following 16 countries reported no current TB cases: Bangladesh, Belize, Bolivia (Plurinational State of), Greece, Hungary, Iran (Islamic Republic of), Israel, Malaysia, New Zealand, Pakistan, Poland, Saudi Arabia, Sudan, Switzerland, Türkiye, Ukraine (see also Methods for definitions).

Anonymised data were available for 453,233 COVID-19 hospital admissions from 62 countries with known TB status (Table 1, Figure 1). In 96%, a SARS-CoV-2 test was reported to be positive. Patient characteristics stratified by TB status are presented in Table 1. Within the sample population, 48% were male, and the median age was 53 years (IQR 38–67). Overall, 8,214 (1.9%) had current TB at the time of hospitalisation, with 46 countries reporting at least one case. A statistically significantly higher proportion of males (2.1%) and patients in the 15–44-year age group (3.4%) had current TB. The severity of COVID-19 also differed according to TB status: 2.1% of those with mild or moderate COVID-19 had current TB, while this proportion was 1.4% among those with severe or critical COVID-19. Current TB was more common among COVID-19 patients coinfected with HIV (5.4% vs. 0.9% in the HIV-negative). Patients with outcomes other than death (palliative discharge, still hospitalised and transfer) were distributed similarly between those with current TB and those without (not shown).

**Table 1.** Characteristics of hospitalised COVID-19 cases on the WHO Global Clinical Platform for COVID-19 by TB status.

	Current TB	No current TB	Total*
	(*n* = 8,214)	(*n* = 435,237)	(*n* = 443,451)
	*n* (%)	*n* (%)	*n*
Sex			
Missing, *n*	7	507	514
Female	3,689 (1.6)	226,681 (98.4)	230,370
Intersex	4 (1.5)	257 (98.5)	261
Male	4,514 (2.1)	207,792 (97.9)	212,306
Age, years			
Missing, *n*	9	1,575	1,584
0–4	156 (0.9)	17,178 (99.1)	17,334
5–14	103 (1.0)	9,966 (99.0)	10,069
15–44	4,261 (3.4)	121,398 (96.6)	125,659
45–64	2,613 (1.6)	161,348 (98.4)	163,961
≥65	1,072 (0.9)	123,772 (99.1)	124,844
Severity			
Missing, *n*	299	6,803	7,102
Mild or moderate	5,291 (2.1)	244,285 (97.9)	249,576
Severe or critical	2,624 (1.4)	184,149 (98.6)	186,773
HIV			
Missing, *n*	2,630	39,558	42,188
Negative	3,054 (0.9)	351,214 (99.1)	354,268
Positive	2,530 (5.4)	44,465 (94.6)	46,995
Chronic cardiac disease			
Missing, *n*	2,310	13,415	15,725
No	5,777 (1.4)	412,761 (98.6)	418,538
Yes	127 (1.4)	9,061 (98.6)	9,188
Diabetes			
Missing, *n*	1,400	11,753	13,153
No	5,661 (1.7)	332,145 (98.3)	337,806
Yes	1,153 (1.2)	91,339 (98.8)	92,492
Hypertension			
Missing, *n*	1,584	8,105	9,689
No	5,072 (1.7)	287,354 (98.3)	292,426
Yes	1,558 (1.1)	139,778 (98.9)	141,336
Chronic pulmonary disease			
Missing, *n*	2,004	8,796	10,800
No	5,192 (1.3)	409,747 (98.7)	414,939
Yes	1,018 (5.7)	16,694 (94.3)	17,712
Asthma			
Missing, *n*	2,000	44,357	46,357
No	5,516 (1.5)	368,887 (98.5)	374,403
Yes	698 (3.1)	21,993 (96.9)	22,691

* 9,782 admissions had missing information on current TB.

### Case-fatality ratios

**Figure 2.**
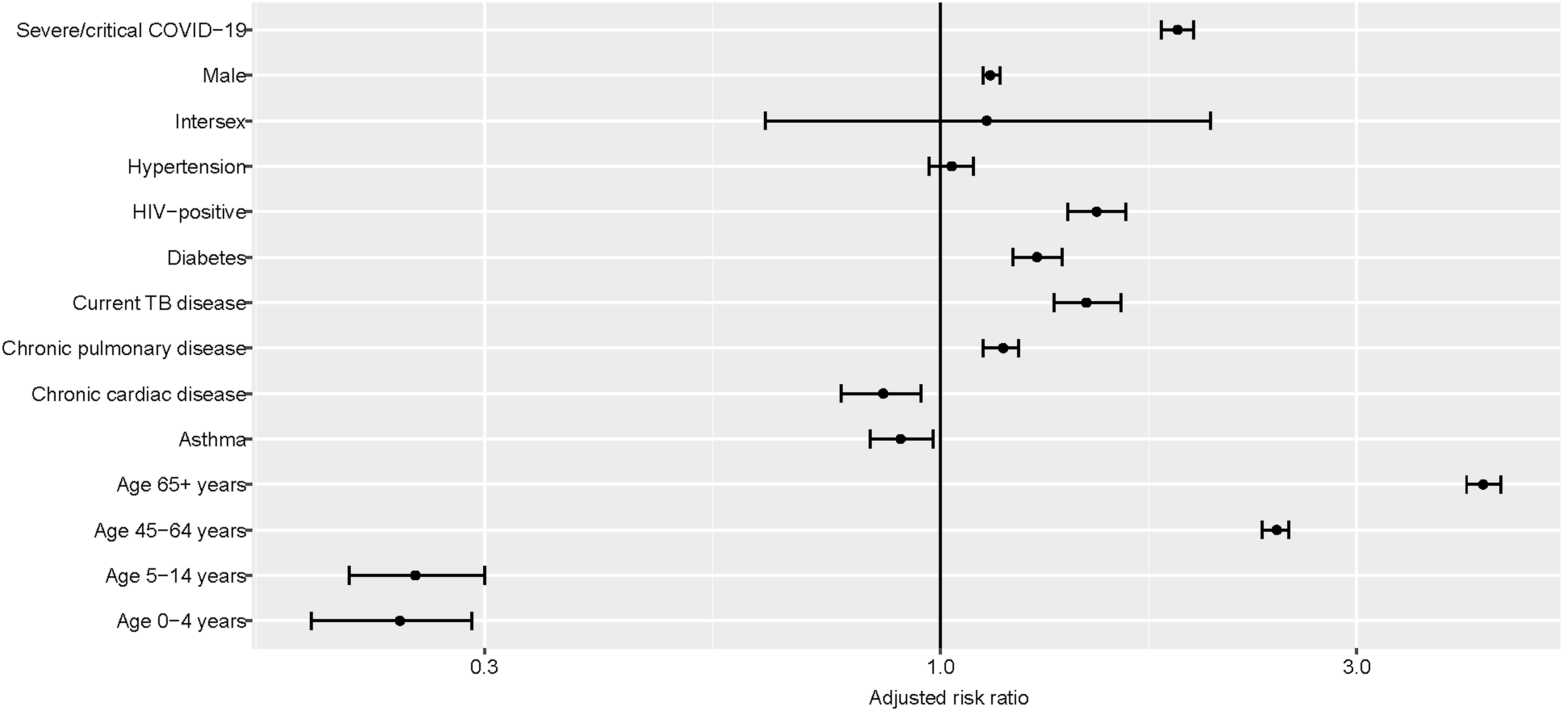
Adjusted risk ratios* of in-hospital mortality among COVID-19 patients on the WHO Global Clinical Platform for COVID-19. *From multivariable log-binomial regression of pooled data, 2020–2023. The following factors were a priori included in the model: age, sex, severity of COVID-19 disease, HIV status, and other comorbidity (chronic cardiac disease, diabetes, hypertension, chronic pulmonary disease, and asthma).

Case-fatality ratios (CFR) are shown in Table 2 and Figure 2, with adjusted risk ratios (aRRs) for in-hospital death. In-hospital CFR was 20.6% (Table 2) and was higher in males than females (22.6% vs. 18.8%; aRR 1.14, 95% CI 1.12–1.17). CFR declined from the last quarter of 2021 (data not shown). CFR increased incrementally with age in adults: 11.3% in 15–44 years, 38.0% in 45–64 years and 50.2% in ≥65 years (aRR in ≥65 years 4.19, 95% CI 4.01–4.39 compared with 15–44 years). Mortality in children was very low (<0.4%). As expected, higher CFR was recorded for severe or critical COVID-19 compared with mild or moderate disease (31.4% vs. 19.3%).

**Table 2.** CFRs and aRRs* of in-hospital mortality by subgroup of COVID-19 patients, WHO Global Clinical Platform for COVID-19.

	CFR	
	*n* (%)	aRR (95% CI)
Overall (*n* = 431,656)^†^	89,087 (20.6)	—
TB disease		
Missing, *n*	817	
Current TB	1,913 (24.5)	1.47 (1.35–1.61)
No current TB	86,357 (20.6)	1
Sex		
Missing, *n*	9	
Female	42,459 (18.8)	1
Intersex	10 (14.1)	1.13 (0.63–2.04)
Male	46,609 (22.6)	1.14 (1.12–1.17)
Age, years		
Missing, *n*	112	
0–4	335 (0.4)	0.24 (0.19–0.29)
5–14	178 (0.2)	0.25 (0.21–0.30)
15–44	10,035 (11.3)	1
45–64	33,784 (38.0)	2.43 (2.34–2.51)
≥65	44,643 (50.2)	4.19 (4.01–4.39)
Severity		
Missing, *n*	323	
Mild or moderate	32,119 (13.1)	1
Severe or critical	56,645 (31.4)	1.87 (1.79–1.95)
HIV		
Missing, *n*	11,200	
Negative	66,878 (19.3)	1
Positive	11,009 (24.3)	1.51 (1.40–1.63)
Chronic cardiac disease		
Missing, *n*	4,168	
No	82,244 (20.2)	1
Yes	2,675 (30.8)	0.86 (0.77–0.95)
Diabetes, *n*		
Missing	2,803	
No	58,710 (17.9)	1
Yes	27,574 (30.8)	1.29 (1.21–1.38)
Hypertension		
Missing, *n*	1,829	
No	47,182 (16.6)	1
Yes	40,076 (29.1)	1.03 (0.97–1.09)
Chronic pulmonary disease		
Missing, *n*	2,552	
No	81,644 (20.2)	1
Yes	4,891 (27.7)	1.18 (1.12–1.23)
Asthma, *n*		
Missing	13,086	
No	71,585 (19.7)	1
Yes	4,416 (19.8)	0.90 (0.83–0.98)

* From multivariable log-binomial regression of pooled data, 2020–2023. The following factors were a priori included in the model: age, sex, severity of COVID-19 disease, HIV status, and other comorbidity (chronic cardiac disease, diabetes, hypertension, chronic pulmonary disease, and asthma).

† 21,577 admissions without information about in-hospital death excluded from the analysis.

CFR = case-fatality ratio; aRR = adjusted risk ratio; CI = confidence interval.

Those coinfected with HIV had a higher likelihood of death (24.3% versus 19.3% among HIV-negative patients), as did those with current TB compared with those without current TB (24.5% vs. 20.6%, respectively). After adjustment, current TB was strongly associated with a higher risk of death (aRR 1.47, 95% CI 1.35–1.61). The inclusion of ‘past TB’ in the model reduced this estimate (aRR 1.26, 95% CI 1.17–1.36). Stratification by HIV status demonstrated a higher relative risk of mortality associated with TB in those with HIV coinfection than those without (aRR 1.36, 95% CI 1.28–1.44 vs. 1.12, 95% CI 1.07–1.16). Apart from TB, other comorbidities increased the risk of death, including diabetes (aRR 1.29, 95% CI 1.21–1.38) and chronic pulmonary disease other than TB (aRR 1.18, 95% CI 1.12–1.23).

### Time to death

The median time to death among all COVID-19 patients was 7 days (IQR 3–14), whereas the median time to discharge was 6 days (IQR 3–10) (Table 3). Time to death was shorter in patients with current TB than those without (5 days, IQR 2–12 vs. 7 days, IQR 3–15; *P* < 0.001). Of patients discharged alive, those with TB had a longer hospitalisation than those without (9 days, IQR 5–15 vs. 6 days, IQR 3–10; P < 0.001).

**Table 3.** Time to discharge alive and time to death among hospitalised COVID-19 cases by TB status, WHO Global Clinical Platform for COVID-19.

	Current TB	No current TB	Total	*P*-value
Time to discharge alive, days, median [IQR]	9 [5–15]	6 [3–10]	6 [3–10]	<0.001
Time to death, days, median [IQR]	5 [2–12]	7 [3–15]	7 [3–14]	<0.001

IQR = interquartile range.

## DISCUSSION

Our analysis of the Global Clinical Platform on COVID-19 explored links between TB and severe or fatal outcomes in hospitalised COVID-19 patients. We found current TB to be an independent risk factor for in-hospital mortality regardless of illness severity at hospital admission and quantified this risk. Among patients dying in the first week after admission, the time to death was shorter in people with current TB.

The potential for SARS-CoV-2 and TB interaction worsening outcomes has been previously suggested. However, a systematic review from 2022 found limited evidence base addressing the impact of TB on COVID-19 death and unfavourable outcomes.^10^ Similarly, a 2023 systematic review of TB and SARS-CoV-2 coinfection demonstrated low quality and conflicting evidence on the effect of immunomodulating treatment on TB outcomes and COVID-19-related outcomes (progression to severe or critical COVID-19 or death).^11^ There were no studies specifically designed to address these questions.

Our subsequent findings suggest that in hospitalised COVID-19 patients, the presence of concurrent TB increases the risk of death by about 50%. Past TB also represents an increased risk. The potential implications for health policy include the prioritisation of COVID-19 vaccination in people with current or past TB. It may also be expedient to test all TB patients for SARS-CoV-2 early on when there is known virus transmission in the community. The quick onset of death after hospitalisation may suggest a need for earlier admission to hospital care, more triage, and close care following admission.

TB remains a major global public health challenge. It is a disease firmly rooted in poverty and associated with a shortage of universal health care. The advent of COVID-19 represented another potential threat to efforts to curb TB. COVID-19 has had a profound impact on global poverty: in 2021, it was estimated that close to 100 million people worldwide were living on less than US$1.90 per day due to the pandemic.^12^ The global epidemic of TB is influenced by different social and health-related factors, particularly undernourishment, HIV infection, alcohol use disorders, smoking, and diabetes, many of which are adversely influenced by poverty. Similarly to people with TB, COVID-19 patients risk poorer outcomes if they are older and have diabetes or HIV.

An earlier study using the WHO Global Clinical Platform analysed records from 38 countries available until July 2021, concluding that HIV was an independent risk factor for both severe COVID-19 at admission and in-hospital mortality.^13^ The findings have informed WHO immunisation policy, which prioritises vaccination for people living with HIV. In our analysis, there was limited effect modification by HIV status, implying that the risk of worse outcomes in COVID-19 patients with TB was present in both people with HIV and those without.

Despite the utility for public health, these data have some limitations. No external data validation was possible after submission beyond coherency checking. Definitions of current and past TB may have differed between countries. Data completion was variable, limiting the analysis particularly concerning factors plausibly, or known to be, associated with the outcomes (e.g., HIV viral load). Potentially useful variables such as CD4 counts, TB diagnostics or treatment used, nutritional status, income, quantification of alcohol intake, diabetes control, and other socioeconomic determinants were not included to keep the CRF brief.

The high proportion of patients with current TB in some cohorts might indicate a reporting bias to the platform (selection or ascertainment bias) and certainly reflect a geographical over-representation of some countries, for example, South Africa, where data collection and submission were extensive. Secular changes such as predominant circulating viral strains, vaccination coverage, and sampling criteria may affect results over time, but we have reported the whole dataset. The absence of information about post-discharge survival may have led to an underestimation of overall mortality, although this would probably be independent of current TB status.

In conclusion, this multi-country individual-level data analysis shows that TB is independently associated with an increased risk of presenting with severe or critical COVID-19 at hospital admission and an increased likelihood of in-hospital mortality. The analysis demonstrates that a unified data collection platform can continuously inform public health action. Within the framework of pandemic preparedness, provisions must be made to strengthen the collection and timely sharing of data with the WHO, including data on health conditions like TB, which are critical drivers of disease burden and death.
